# Supplementary material for: Content-rich biological network constructed by mining PubMed abstracts
Source: BMC Bioinformatics. 2004 Oct 8;5:147. doi: 10.1186/1471-2105-5-147 (PMC528731; doi:10.1186/1471-2105-5-147)
Supplement: Additional File 2 — The original results of the above study (non-essential files are deleted to keep the file size under the limit set by BMC bioinformatics). [file 1471-2105-5-147-S2.bz2 › chilibotAdditionalFile2/dip20/2ID9194558E2/html/BCL2_RAF1.html]

 


 **BCL2** and **RAF1** 
  
Found 126 abstracts in PubMed, retrieved 20.  
 

 What does Google say? 
 PDF only 
| .edu only 

---

**Interactive relationship** (e.g. stimulation, inhibition, etc)

**Inhibitory relationship**- Results Transfection of the myeloid leukemia cell lines with dsRNA corresponding to c raf  [ **RAF1** ]  and bcl 2  [ **BCL2** ]  genes decreased the expression of Raf 1  [ **RAF1** ]  and Bcl 2  [ **BCL2** ]  proteins.  Ref: 12536201 Cancer Gene Ther, 2003
- Finally, transient transfection of a dominant negative Ras allele induced Bcl 2  [ **BCL2** ]  expression and reduced Bcl 2  [ **BCL2** ]  Raf 1  [ **RAF1** ]  association demonstrating a requirement for Ras.  Ref: 12488548 Mol Pharmacol, 2003
- trans Resveratrol reverses phosphorylation of Bcl 2  [ **BCL2** ]  induced by paclitaxel and concomitantly blocks Raf 1  [ **RAF1** ]  phosphorylation, also observed after paclitaxel exposure, thus suggesting that Bcl 2  [ **BCL2** ]  inactivation may be dependent on the activation of the Raf  [ **RAF1** ]  Ras cascade.  Ref: 12510025 Neurochem Int, 2003
- FDCP Mix BCL 2  [ **BCL2** ]  cells grown in IL 3 were distinguished from FDCP Mix and FDCP Mix BCL X L cells by a striking reduction in cellular levels of Raf 1  [ **RAF1** ]  protein.  Ref: 12721288 J Biol Chem< MedlineTA>J Biol Chem, 2003
**Neutral relationship**- Replacement of the BCL 2  [ **BCL2** ]  BH4 domain with the related BCL X L BH4 sequence resulted in a switch of FDCP Mix BCL 2  [ **BCL2** ]  to erythroid fate accompanied by persistence of Raf 1  [ **RAF1** ]  protein expression.  Ref: 12721288 J Biol Chem< MedlineTA>J Biol Chem, 2003
- Taken together, these findings show that increased expression of p27 and Bcl 2  [ **BCL2** ]  is concomitant with altered association between Ras, Raf 1  [ **RAF1** ]  and Bcl 2  [ **BCL2** ]  and suggest that this is responsible for the growth inhibitory properties of FTI 277.  Ref: 12488548 Mol Pharmacol, 2003
- These effects are associated with differential regulation of Raf 1  [ **RAF1** ]  expression, perhaps involving the previously identified interaction between BCL 2  [ **BCL2** ]  BH4 and the catalytic domain of Raf 1  [ **RAF1** ] .  Ref: 12721288 J Biol Chem< MedlineTA>J Biol Chem, 2003

**Non-interactive relationship** (e.g. studied together, co-existance, homology, etc.)

- In the absence of doxorubicin, overexpression of Bcl 2  [ **BCL2** ]  or a constitutively active form of Raf 1  [ **RAF1** ]  in MCF 7 cells did not affect proliferation rate.  Ref: 12631622 Clin Cancer Res, 2003
- Combined RNAi for c raf  [ **RAF1** ]  and bcl 2  [ **BCL2** ]  induced apoptosis in HL 60, U937, and THP 1 cells and increased chemosensitivity to etoposide and daunorubicin.  Ref: 12536201 Cancer Gene Ther, 2003
- Using U937 leukemic cells, we evaluated the effect of docetaxel on phosphatidylcholine PC and its metabolites, phosphatidic acid PA and diacylglycerol DAG, and their impact on MAPK and NF kappa B activation, as well as on Raf 1  [ **RAF1** ]  and Bcl 2  [ **BCL2** ]  phosphorylation.  Ref: 12724857 J Exp Ther Oncol< MedlineTA>J Exp Ther Oncol, 2003
- In addition, increased phosphorylation of Raf  [ **RAF1** ] , MEK1 2 and Bcl 2  [ **BCL2** ]  was observed in MAD2 overexpressing cells in response to vincristine.  Ref: 12527913 Oncogene, 2003
- These observations suggest both independent and overlapping roles for Raf 1  [ **RAF1** ]  and Bcl 2  [ **BCL2** ]  oncogenes in the resistance to growth inhibition by doxorubicin.  Ref: 12631622 Clin Cancer Res, 2003
- Although the state of 17 AAG mediated decline in Akt, c Raf  [ **RAF1** ]  1 and c Src protein levels was blunted, the totalt decline was not compromised in HL 60 Bcl 2  [ **BCL2** ]  and HL 60 Bcl xL cells.  Ref: 12623837 Blood< MedlineTA>Blood, 2003
- Here we show that apoptosis triggered by Raf  [ **RAF1** ]  depletion cannot be overcome by ectopic Bcl 2  [ **BCL2** ]  expression and occurs in the absence of cytochrome c release, arguing against a direct impact of c Raf  [ **RAF1** ]  on mitochondrial pathways of apoptosis regulation.  Ref: 12584738 Int J Cancer, 2003
- Moreover, increased expression of Bcl 2  [ **BCL2** ]  protein was observed in HepG2 and Huh7 cells treated with FTI 277, and this was coincidental with reduced association between Raf 1  [ **RAF1** ]  and Bcl 2  [ **BCL2** ] .  Ref: 12488548 Mol Pharmacol, 2003
- One antisense compound has been approved for local treatment of cytomegalovirus induced retinitis, and several others are in clinical trials, including those targeting the mRNA of Bcl 2  [ **BCL2** ] , protein kinase C alpha PKC alpha, c raf  [ **RAF1** ]  or Ha ras.  Ref: 12867066 Lung Cancer, 2003
- Regulation of 17 AAG induced apoptosis role of Bcl 2  [ **BCL2** ] , Bcl xL, and Bax downstream of 17 AAG mediated downregulation of Akt, Raf 1  [ **RAF1** ]  and Src kinases.  Ref: 12623837 Blood< MedlineTA>Blood, 2003
- Methods Four myeloid leukemia cell lines HL 60, U937, THP 1, and K562 were transfected with dsRNA duplexes corresponding to the endogenous c raf  [ **RAF1** ]  and bcl 2  [ **BCL2** ]  genes and the gene expression inhibition was assessed.  Ref: 12536201 Cancer Gene Ther, 2003
- Cryptophycin 52 induced phosphorylation of c  **raf1**  and bcl 2  [ **BCL2** ]  and or bcl x to comparable levels in all cell lines studied, and LNCaP cells overexpressing bcl 2  [ **BCL2** ]  were more resistant to cryptophycin 52 induced apoptosis.  Ref: 12473608 Clin Cancer Res, 2002
- Growth Inhibition by the Farnesyltransferase Inhibitor FTI 277 Involves Bcl 2  [ **BCL2** ]  Expression and Defective Association with Raf 1  [ **RAF1** ]  in Liver Cancer Cell Lines.Farnesyltransferase inhibitors block the growth of tumor cells in vitro and in vivo with minimal toxicity toward normal cells.  Ref: 12488548 Mol Pharmacol, 2003
- Induction of Raf 1  [ **RAF1** ]  activity in MCF 7 cells overexpressing Bcl 2  [ **BCL2** ]  resulted in greater doxorubicin resistance than induction of Raf 1  [ **RAF1** ]  activity in MCF 7 cells lacking Bcl 2  [ **BCL2** ]  overexpression.  Ref: 12631622 Clin Cancer Res, 2003
- In this study we investigated effects of Bcl 2  [ **BCL2** ]  and Raf 1  [ **RAF1** ]  on doxorubicin induced growth inhibition of MCF 7 breast cancer cells.  Ref: 12631622 Clin Cancer Res, 2003
- MAD2 induced sensitization to vincristine is associated with mitotic arrest and Raf  [ **RAF1** ]  Bcl 2  [ **BCL2** ]  phosphorylation in nasopharyngeal carcinoma cells.  Ref: 12527913 Oncogene, 2003
- Moreover, enforced expression of Raf 1  [ **RAF1** ]  redirected FDCP Mix BCL 2  [ **BCL2** ]  cells to an erythroid fate, and prohibited generation of myeloid cells.  Ref: 12721288 J Biol Chem< MedlineTA>J Biol Chem, 2003
- Raf 1  [ **RAF1** ]  and bcl 2  [ **BCL2** ]  induce distinct and common pathways that contribute to breast cancer drug resistance.  Ref: 12631622 Clin Cancer Res, 2003
- Conclusions RNAi is a functional pathway in human myeloid leukemia cell lines and combined RNAi of c raf  [ **RAF1** ]  and bcl 2  [ **BCL2** ]  genes may represent a novel approach to leukemia, providing a means to overcome the resistance to chemotherapeutic agents and ultimately to augment the efficacy of chemotherapy in myeloid leukemia.  Ref: 12536201 Cancer Gene Ther, 2003
